# Three Key Aspects of Electron Transfer Behavior in Single-Electrode Triboelectric Nanogenerators for Sensing Optimization

**DOI:** 10.3390/s26010056

**Published:** 2025-12-21

**Authors:** Dazheng Shi, Jingkai Xi, Yu Hou, Siyu Qu, Ding Li

**Affiliations:** 1 Beijing Key Laboratory of High-Entropy Energy Materials and Devices, Beijing Institute of Nanoenergy and Nanosystems, Chinese Academy of Sciences, Beijing 101400, China; shidazheng@binn.cas.cn (D.S.); xijingkai@binn.cas.cn (J.X.); houyu@binn.cas.cn (Y.H.); qusiyu@binn.cas.cn (S.Q.); 2 School of Nanoscience and Engineering, University of Chinese Academy of Sciences, Beijing 100049, China

**Keywords:** electron transfer, polymer functional group, corona polarization, TENG, sensing

## Abstract

With the rapid development of the Internet of Things, self-powered sensing technology has become a crucial solution for scenarios where an external power supply is inconvenient or unavailable, such as wild monitoring and flexible wearables. The triboelectric nanogenerator (TENG)—an excellent self-powered sensor, particularly in the single-electrode mode—demonstrates broad application prospects due to its simple structure and ease of integration. However, a comprehensive understanding of the electron transfer behavior of TENGs for performance optimization remains insufficient. Here, we investigate such behaviors from three key aspects—the polymer functional groups, the configurations of TENGs, and corona polarization. It is found that polymer functional groups critically determine electron transfer ability, with fluorinated polymers exhibiting superior performance across all configurations. Moreover, the configuration significantly influences electron transfer efficiency, where the sliding configuration vastly outperforms contact–separation configurations. Furthermore, the effect of corona polarization is highly configuration-dependent, improving performance in contact–separation configurations while generally reducing it in sliding configuration. These findings provide valuable theoretical guidance and practical strategies for optimizing the design and selecting appropriate materials and configurations of TENG-based self-powered sensors. They also pave the way for a new generation of highly efficient, miniaturized, and adaptive self-powered systems.

## 1. Introduction

From the Industrial Revolution to the Information Age, humanity’s ability to adapt to the physical world has continuously deepened. Precise and efficient sensing technologies have become a key driving force in the process of societal intelligentization. As the perceptual terminals of Internet of Things systems, the performance of sensors directly determines the response speed and decision-making accuracy of the entire information system. A variety of sensing technologies have been developed and applied, including optical fiber sensors [1], laser sensors [2], quantum sensors [3], and various semiconductor sensors [4], in order to measure physical and chemical parameters such as temperature [5,6], humidity [7], pressure [8,9], and gas concentration [10]. However, most conventional sensors rely on external power sources, which poses challenges for their deployment in scenarios such as field monitoring [11], implantable medical devices [12], and flexible wearable electronics [13,14], where power is unavailable or mobility is required. Furthermore, they suffer from bottlenecks such as high maintenance costs, complex structures, and poor environmental adaptability [15].

To overcome these challenges, self-powered sensing technology has gradually become a research hotspot. Originating in 2006, nanogenerators, as an emerging mechanical-to-electrical energy conversion device [16], have not only extended the application of contact electrification to micro/nano energy [17,18,19] and ocean blue energy [20,21,22,23] and promoted in-depth research on the mechanism of contact electrification [24,25,26,27], but have also served as excellent self-powered sensors [28,29,30,31,32,33,34,35]. Among the various working modes of triboelectric nanogenerators (TENGs), the single-electrode mode shows broad prospects in the fields of energy harvesting and dynamic sensing due to its simple structure, ease of integration, and fewer constraints on moving parts [36,37]. However, since the core operating principle of TENGs is contact electrification and electrostatic induction, the performance of TENGs—especially the signal quality when used as sensors—is largely limited by the electron transfer efficiency at the contact electrification interface. Therefore, a deep understanding and effective regulation of the interfacial electron transfer process are crucial for enhancing TENG performance.

To improve interfacial electron transfer efficiency, previous studies have investigated the selection of triboelectric materials, among which polymer materials are particularly critical due to their diverse functional groups and tunable electronic structures [27]. For single-electrode TENGs, prior research has explored the influence of different structural parameters on electron transfer [37]. Additionally, corona polarization has been proven to be an effective method for enhancing the electron transfer efficiency of TENGs. It can significantly improve their triboelectric performance by modulating the surface charge state and molecular group orientation of polymers [38]. However, most existing studies have investigated these factors in an isolated manner, lacking a unified framework to understand the complex interplay among polymer functional groups, configurations of single-electrode TENGs, and corona polarization. This is particularly evident in the electron transfer behavior of polymers with different functional groups across the three common single-electrode TENG configurations—planar contact–separation, curved contact–separation, and sliding configuration—as well as in the varying effects of corona polarization across these different TENG configurations. The unique contribution of this work lies in its systematic and comparative investigation of these three key aspects. It aims to establish a comprehensive understanding of their synergistic effects on electron transfer, thereby providing a new paradigm for the rational design of TENG-based sensors.

Based on these, this study selected eight common polymers covering non-halogenated, chlorinated, and fluorinated groups with different carbon-to-fluorine ratios and molecular configurations, including polyethylene (PE), polypropylene (PP), polyvinyl alcohol (PVA), polyvinyl chloride (PVC), ethylene tetrafluoroethylene (ETFE), polytetrafluoroethylene (PTFE), fluorinated ethylene propylene (FEP), and polychlorotrifluoroethylene (PCTFE), (detailed in Table 1) to investigate their electron transfer behaviors in the three common single-electrode TENG configurations: the planar contact–separation, curved contact–separation, and sliding configurations. The influence of corona polarization on these polymers in the three configurations was further explored. The remainder of this manuscript is organized as follows: Section 2 details the materials and methods used in this study. Subsequently, Section 3.1 establishes the working principles of the three TENG configurations, Section 3.2 details their corresponding electrical output signals, and Section 3.3 statistically compares their performance. Based on these findings, Section 3.4 proposes the underlying mechanism for the observed phenomena. Section 3.5 systematically discusses the electron transfer characteristics of the eight polymer films across the three configurations, while Section 3.6 extends this analysis to the effects of corona polarization. Then, Section 4 discusses the implications of the findings and suggests future research directions. Finally, the key findings are summarized in Section 5, where their implications for optimizing TENG-based sensors are also discussed. Through a comprehensive analysis combining experiments and theoretical investigations, we find that polymers containing highly electronegative functional groups perform excellently in all three configurations. The carbon-to-fluorine ratio and molecular configuration significantly influence electron transfer efficiency. We further elaborate that, due to continuous electron cloud overlap, the sliding configuration exhibits higher electron transfer efficiency than the two contact–separation configurations. Due to the influence of curvature on surface state energy levels, the curved contact–separation configuration shows higher efficiency than the planar contact–separation configuration. We also clarify that the effect of corona polarization strongly depends on the configuration, generally enhancing performance in contact–separation configurations but mostly suppressing it in the sliding configuration. When material selection is limited, the performance of TENG-based self-powered sensors can be effectively compensated for by optimizing the configuration or applying polarization. These findings provide a clear decision-making pathway for the design of self-powered sensors, thereby offering key theoretical and practical foundations for building high-performance, low-cost sensing systems. They also provide a universal strategy for optimizing TENG-based sensors, potentially impacting fields such as human–machine interfaces and IoT sensing.

## 2. Materials and Methods

### 2.1. Experimental Materials

Table 1 shows the specifications of the polymer films used in this study.

**Table 1:** Specifications of the polymer films used in this study.

Material (Abbreviation)	Thickness (µm)	Supplier
Polyethylene (PE)	300	Shunmao Plastic Co., Ltd. (Shenzhen, Guangdong, China)
Polypropylene (PP)	300	Shunmao Plastic Co., Ltd. (Shenzhen, Guangdong, China)
Polyvinyl alcohol (PVA)	80	Li Yun Factory Store (Suzhou, Jiangsu, China)
Polyvinyl chloride (PVC)	100	Shunmao Plastic Co., Ltd. (Shenzhen, Guangdong, China)
Ethylene tetrafluoroethylene copolymer (ETFE)	50	Weitelang Technology Co., Ltd. (Shanghai, China)
Polytetrafluoroethylene (PTFE)	50	Colleague Hardware Flagship Store(Suzhou, Jiangsu, China)
Fluorinated ethylene propylene copolymer (FEP)	50	Weitelang Technology Co., Ltd. (Shanghai, China)
Polychlorotrifluoroethylene (PCTFE)	50	Weitelang Technology Co., Ltd. (Shanghai, China)

### 2.2. Fabrication of TENGs as Probes for Electron Transfer Investigation

**Figure 1:**
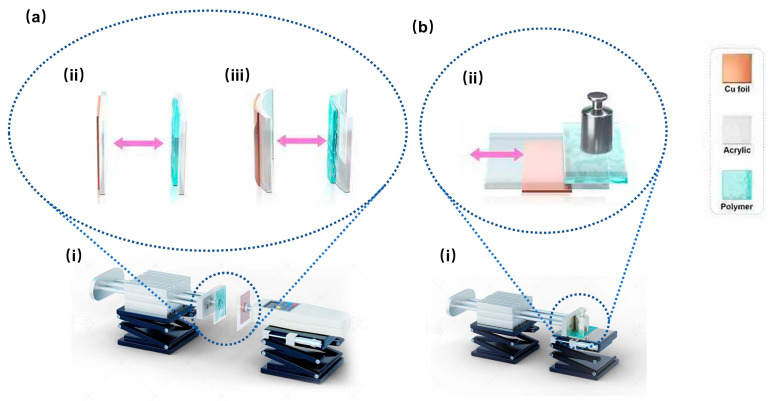
Schematic illustrations of triboelectric nanogenerator device structures and testing methods. (**a**) Contact–separation configuration: (**i**) schematic of the testing setup, (**ii**) schematic of the planar structure, (**iii**) schematic of the curved structure. (**b**) Sliding configuration: (**i**) schematic of the testing setup, (**ii**) schematic of the sliding structure. Purple arrows indicate the direction of relative motion.

For the planar contact–separation configuration, as illustrated in Figure 1a(ii), the contact–separation electrification system comprised a pair of triboelectric layers with opposite electrification properties: a rectangle-shaped 4 cm (in width) × 7 cm (in length) polymer film and a rectangle-shaped 8 cm (in width) × 8 cm (in length) acrylic plate. The polymer film was affixed to another rectangle-shaped 5.4 cm (in width) × 8 cm (in length) planar acrylic substrate using double-sided tape. Meanwhile, a rectangle-shaped 3 cm (in width) × 7 cm (in length) double-sided adhesive conductive copper foil was adhered to the rear surface of the acrylic plate, serving as the electrode. Electrical wires were embedded between the copper foil and the acrylic substrate, connecting to a Keithley 6514 electrometer system (Keithley Instruments, LLC, Solon, Ohio, United States of America) for electrical characterization. All the main components were aligned at their centers. The effective electron transfer probing area matched the 3 cm (in width) × 7 cm (in length) copper foil due to electrostatic induction, which will be further explained in Section 3.

For the curved contact–separation configuration, as illustrated in Figure 1a(iii), the electrification interface was established between two conformally curved components: a polymer film of the same size as described above, and the concave surface of a curved acrylic plate (detailed in Table 2). The polymer film was affixed via double-sided tape to the convex surface of another identical curved acrylic plate, serving as the substrate. On the convex side of the curved acrylic plate used for electrification, a 3 cm (in width) × 7 cm (in length) double-sided adhesive conductive copper foil was attached to serve as the electrode, with embedded wires connecting it to a Keithley 6514 electrometer system for electrical characterization. All the main components were aligned at their centers. The effective electron transfer probing area was the same as described above.

For the sliding configuration, as shown in Figure 1b(ii), the sliding-configuration triboelectric system used the same sizes of polymer film, acrylic plates, and copper foil as described for the planar contact–separation surfaces. However, all of these materials were aligned along the length direction. All three surfaces had the same effective electron transfer probing area as the copper foil.

### 2.3. Characterization of Electron Transfer Using TENG as a Probe

The relative motion between the two triboelectric layers in all TENGs was precisely controlled using a LinMot E1100 linear motor system (NTI AG, Spreitenbach, Zurich, Switzerland). The electrical output performance was characterized by a Keithley 6514 electrometer.

For the contact–separation configuration (Figure 1a(i)), a rectangular polymer film triboelectric layer, fixed on an acrylic substrate, was vertically mounted and attached to the actuator of the linear motor via double-sided tape. The rectangular acrylic plate, serving as the other triboelectric layer, was aligned vertically to the polymer film on the opposite side and installed on a Handpi SH-10N digital force gauge (Yueqing H&P Instruments Co., Ltd., Yueqing, Zhejiang, China). The linear motor was programmed to operate at a speed of 1 m/s with a maximum separation distance of 8 cm between the two layers. A dwell time of 1 s was set both after contact and at the maximum separation distance. The force gauge continuously monitored the contact force to maintain a constant maximum pressure of 4 N during each contact event.

For the sliding configuration (Figure 1b(i)), one triboelectric layer—polymer film—was placed in a horizontal orientation. The acrylic substrate holding the polymer film was attached with double-sided tape to a 4 N counterweight, which was then fixed to the actuator. The other triboelectric layer—a rectangular acrylic plate—was horizontally mounted on the workbench using double-sided tape. The linear motor was programmed to traverse a total distance of 8 cm at a speed of 1 m/s, with a dwell time of 1 s at both ends of the motion. The 4 N counterweight was used to maintain a consistent normal force of 4 N between the triboelectric layers, ensuring comparability with the contact–separation configuration. Prior to formal recording for both TENG configurations, the triboelectric layers reached a steady state through charge accumulation from repeated contact electrification, controlled by the linear motor.

The comparative study of the three configurations was designed to evaluate the impact of contact geometry and motion type on electron transfer. To ensure a direct comparison, key parameters, including a 4 N contact force, 1 m/s driving speed, and identical material dimensions and effective contact areas, were kept constant across all configurations. This approach ensured that the observed differences in electrical output were primarily due to the inherent differences among the planar contact–separation, curved contact–separation, and sliding mechanisms.

## 3. Results

### 3.1. Working Principles of the Three Configurations

According to the triboelectric series, in all three electrification systems, electrons were transferred from the acrylic plate triboelectric layer to the polymer film triboelectric layer. As a result, the polymer film triboelectric layer became negatively charged, while the acrylic plate triboelectric layer became positively charged.

**Figure 2:**
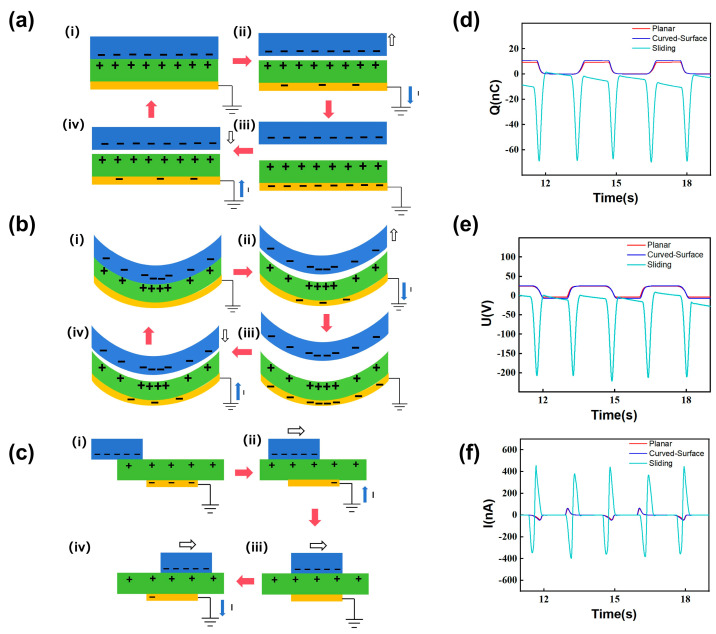
Working principles of the three configurations and their electrical outputs. The working principles of (**a**) planar contact–separation configuration, (**b**) curved contact–separation configuration, and (**c**) sliding configuration, with their corresponding electrical outputs: (**d**) transferred charge, (**e**) open-circuit voltage, and (**f**) short-circuit current. It is clear that the electrode interface curvature has little effect on the electrical signal shape of the contact–separation configuration, but switching to sliding configuration causes a significant change in the signal shape. In this figure, the blue solid arrows indicate the direction of current flow, the black hollow arrows indicate the direction of relative motion, and the red solid arrows indicate the procedural sequence. Subfigures (**i**–**iv**) in panels (**a**,**b**) correspond to the stages of contact, separating motion, maximum separation, and approaching motion, respectively. Subfigures (**i**–**iv**) in panel (**c**) correspond to the stages where the sliding film is at the leftmost position (no overlap), slides to the right and begins to overlap the electrode, fully covers the electrode, and slides beyond the electrode, respectively.

In the contact–separation configuration systems, upon contact, as shown in Figure 2a(iii),b(iii), electrons were transferred from the acrylic plate triboelectric layer to the polymer film triboelectric layer according to the triboelectric series. As a result, the polymer film triboelectric layer became negatively charged, while the acrylic plate triboelectric layer became positively charged. Then, as illustrated in Figure 2a(iv),b(iv), the linear motor separated the two layers gradually. This separation increased the induced negative charge on the copper foil due to electrostatic induction, generating a current flow from the copper foil to the ground. When the maximum separation distance of 8 cm (between the polymer film triboelectric layer and the acrylic plate triboelectric layer) was reached, as shown in Figure 2a(i),b(i), the maximum amount of negative charge was induced on the copper foil electrode. Subsequently, as depicted in Figure 2a(ii),b(ii), the polymer film triboelectric layer was driven toward the acrylic plate triboelectric layer by the linear motor. This motion resulted in a reduction in the induced negative charge on the copper foil, accompanied by a current flow from the ground to the copper foil. Finally, the relative distance returned to its maximum, completing one cycle and initiating the next.

For the sliding-configuration system, as illustrated in Figure 2c(i), when the negatively charged polymer film triboelectric layer was positioned on the left side of the acrylic plate triboelectric layer with no vertical overlap with the copper foil electrode, the maximum amount of negative charge was induced on the copper foil electrode due to electrostatic induction from the positively charged acrylic plate triboelectric layer. Subsequently, as shown in Figure 2c(ii)–c(iv), the polymer film triboelectric layer was driven by the linear motor to slide from the left side to the right side. During this motion, it maintained continuous contact with the acrylic plate triboelectric layer and began to overlap with the copper foil electrode vertically. This relative movement resulted in a reduction in the induced negative charge on the copper foil compared to the initial state, and the current flowed from the ground to the copper foil (Figure 2c(ii)). When the polymer film triboelectric layer fully covered the copper foil vertically (Figure 2c(iii)), there was no further electron transfer at this moment, and the current flow presented as zero. When the polymer film triboelectric layer moved further to the right portion of the copper foil (Figure 2c(iv)), electrons flowed from the ground to the copper foil, reversing the current flow direction compared with the situation in Figure 2c(ii). Eventually, the polymer film triboelectric layer reached the right end of the acrylic plate triboelectric layer and came to a stop. Subsequently, it began to move leftward—a process that can be regarded as the reverse of the above description.

### 3.2. Electrical Output Signals and Analysis

Figure 2d–f show the measured transferred charge (*Q*_sc_), open-circuit voltage (*V*_oc_), and short-circuit current (*I*_sc_) for electrification systems with FEP-acrylic in three configurations as functions of time during the repeated motions described above, respectively. The red, dark blue, and light blue curves represent the electrical signals of the planar contact–separation configuration system, the curved contact–separation configuration system, and the sliding configuration system, respectively. In the figures, all three electrical signals are aligned from state (i) in Figure 2a–c for better comparison, for instance, at time t = 12 s. For the two contact–separation configurations shown in Figure 2a,b, the values of *Q*_sc_, *V*_oc_, and *I*_sc_ were set to zero, as described in their working principles above (state (i)). For the sliding configuration system shown in Figure 2c, the values of *Q*_sc_, *V*_oc_, and *I*_sc_ were set to zero, as described in its working principles above (state (i)).

For the contact–separation configuration systems of the planar structure and curved structure, their signal profiles show no significant differences and can therefore be discussed collectively. As shown in Figure 2d–f, when the systems were in the initial states illustrated in Figure 2a(i),b(i), *Q*_sc_, *V*_oc_, and *I*_sc_ were zero. When the systems reached the states depicted in Figure 2a(ii),b(ii), *Q*_sc_ and *V*_oc_ started to increase with the separation, and *I*_sc_ became positive as the electrons moved from the ground to the electrodes due to electrostatic induction. In the states illustrated in Figure 2a(iii),b(iii), *Q*_sc_ and *V*_oc_ reached their maximum values, while *I*_sc_ remained at zero. As the systems transitioned to the states illustrated Figure 2a(iv),b(ii), both *Q*_sc_ and *V*_oc_ began to decrease as the layers approached, and *I*_sc_ became negative as the electrons moved from the electrodes to the ground due to electrostatic induction.

For the sliding configuration system, when it was in the initial state shown in Figure 2c(i), *Q*_sc_, *V*_oc_, and *I*_sc_ all remained near zero. As the system entered the state shown in Figure 2c(ii), both *Q*_sc_ and *V*_oc_ decreased, and *I*_sc_ became negative. When the system reached the state in Figure 2c(iii), *Q*_sc_ and *V*_oc_ reached their minimum values, and *I*_sc_ returned to zero. When the system reached the state in Figure 2c(iv), *Q*_sc_ and *V*_oc_ increased, and *I*_sc_ became positive. The film then reached the rightmost position and began to move backward. The electrical signal variations during this return motion are generally consistent with those described above.

### 3.3. Investigation of Output Characteristics for Three-Configuration Systems

**Figure 3:**
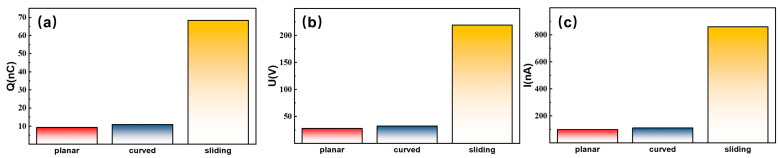
Statistical comparison of (**a**) the transferred charge (*Q*_sc_), (**b**) the open-circuit voltage (*V*_oc_), and (**c**) the short-circuit current (*I*_sc_) for electrification systems with FEP-acrylic in three configurations. It can be clearly observed that the magnitudes of Q, U, and I for the sliding configuration are significantly greater than those of the two contact–separation configurations, while the curved contact–separation configuration yields slightly higher electrical outputs than its planar counterpart.

As clearly illustrated in Figure 3a–c, the most prominent feature of the electrical output performance for electrification systems with FEP-acrylic among the three triboelectric systems—planar contact–separation, curved contact–separation, and sliding configuration—is the significantly higher electrical signals generated by the sliding configuration system compared to the two contact–separation configurations. The measurements of *Q*_sc_, *V*_oc_, and *I*_sc_ demonstrate distinct performance differences across these configurations. The planar contact–separation configuration yielded the lowest output, with *Q*_sc_ ≈ 9.3 nC, *V*_oc_ ≈ 28.3 V, and *I*_sc_ ≈ 101.13 nA. In contrast, the sliding configuration device performed significantly better, with *Q*_sc_ ≈ 68.27 nC, *V*_oc_ ≈ 219.2 V, and *I*_sc_ ≈ 858.8 nA, which are approximately 7.3, 7.7, and 8.5 times higher than those of the planar contact–separation configuration in terms of *Q*_sc_, *V*_oc_, and *I*_sc_, respectively. The curved contact–separation configuration exhibited slightly higher performance than the planar one, achieving values of *Q*_sc_ ≈ 11.0 nC, *V*_oc_ ≈ 32.8 V, and *I*_sc_ ≈ 112.1 nA. 

### 3.4. Proposed Mechanism

**Figure 4:**
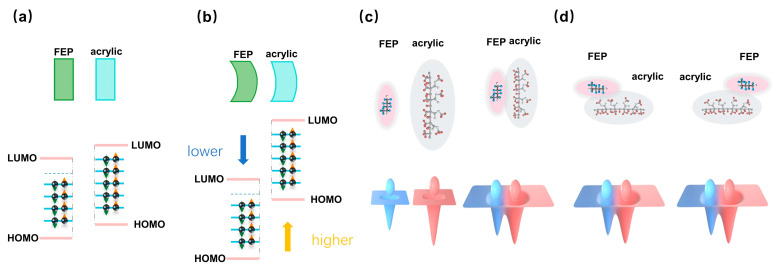
Mechanism of higher electrical output in curved interfaces compared to planar ones and in sliding configuration compared to contact–separation configuration. Surface state model of FEP and acrylic for (**a**) ideally planar surfaces and (**b**) curved surfaces. The curvature of the interface increases the energy level difference between the two materials, thereby facilitating more efficient electron transfer. (**c**) Molecular schematic and corresponding energy level diagram for FEP and acrylic in (**c**) contact–separation and (**d**) sliding configuration. In sliding configuration, the prolonged duration of electron cloud overlap provides greater opportunity for electron transfer compared to the brief contact in contact–separation configuration.

Based on the experimental results, possible mechanisms are proposed. As shown in Figure 4a, when the rectangular FEP film and the acrylic plate come into contact, electrons transfer from the acrylic plate to the FEP film due to the lower neutral level (E_n_) of the FEP, resulting in the FEP being negatively charged and the acrylic being positively charged after contact electrification.

As shown in Figure 4b, when the contact surfaces are curved, the neutral level of the convex FEP surface shifts downward relative to that of a planar surface, while that of the concave acrylic surface shifts upward. This curvature-induced shift in the neutral levels for identical materials enlarges their difference between the FEP and acrylic surfaces compared to the planar case. The increased difference in the neutral levels facilitates greater electron transfer, leading to a higher transferred charge [39].

As shown in Figure 4c, during the contact–separation motion between the FEP film and the acrylic plate, electron cloud overlap at the atomic level occurs briefly at the moment of contact, enabling limited electron transfer, according to the electron cloud-potential well model. Upon separation, the electron clouds cease to overlap, terminating further electron transfer. In contrast, during sliding motion, the electron clouds remain in a state of continuous overlap at the contact interface, providing prolonged opportunities and additional pathways for electron transfer. Consequently, the sliding configuration results in a greater number of electrons being transferred compared to the contact–separation configuration, thereby generating a higher transferred charge. 

### 3.5. Electron Transfer Characteristics of Eight Polymer Films in Three Configurations

**Figure 5:**
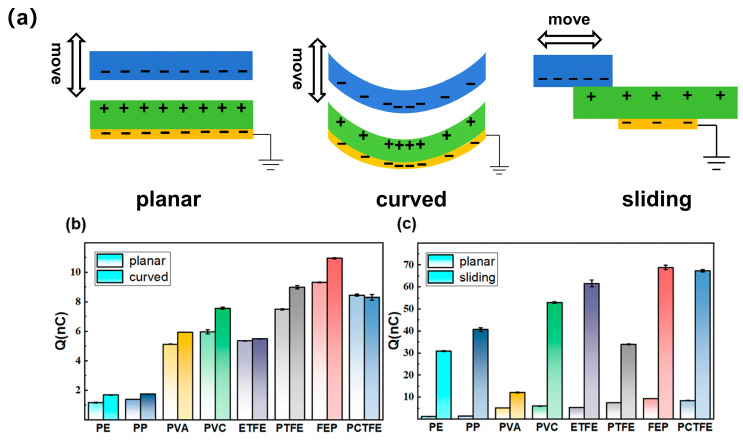
(**a**) Schematic diagram illustrating the three configurations of the single-electrode TENG. Statistical comparisons of transferred charge (*Q*_sc_) for different polymer films in two cases: (**b**) Planar and curved contact–separation configurations, where the light-colored bars on the left and the dark-colored bars on the right represent the planar and curved interfaces, respectively. The curved interface provides a consistent enhancement over the planar one in contact–separation. (**c**) Planar contact–separation and sliding configurations, where the light-colored bars on the left and the dark-colored bars on the right represent the planar contact–separation and sliding configurations, respectively. The sliding configuration exhibits a superior electron transfer capability compared to both contact–separation configurations.

To further investigate these phenomena and validate the proposed explanation, we explored the electron transfer situation when seven other polymers were used to replace FEP in the triboelectric system. These polymers included polyethylene (PE), polypropylene (PP), polyvinyl alcohol (PVA), polyvinyl chloride (PVC), ethylene tetrafluoroethylene copolymer (ETFE), polytetrafluoroethylene (PTFE), and polychlorotrifluoroethylene (PCTFE). The selected polymer films are commonly used and commercially available. Among them, PP, PVA, PVC, and PTFE were chosen for comparison with PE to study the effect of substituting hydrogen atoms (-H) on the carbon chain with methyl (-CH_3_), hydroxyl (-OH), chlorine (-Cl), and fluorine (-F) functional groups, respectively. ETFE, PTFE, and FEP were selected to examine the influence of different carbon-to-fluorine ratios and different molecular configurations, when the carbon-to-fluorine ratios are the same, on electron transfer. PCTFE was also included to investigate the effect of having both fluorine and chlorine functional groups. The schematic diagrams of the three configurations investigated in this section are presented in Figure 5a. The corresponding measured transferred charges for the eight polymers are summarized in Figure 5b, c.

In the planar contact–separation configuration, the electronegativity of the polymer functional groups is a key factor affecting the amount of electron transfer, as shown in Figure 5b. Importantly, due to the significantly higher electronegativity of fluorine (electronegativity 4.0) and chlorine (electronegativity 3.0) compared to carbon (electronegativity 2.5) and hydrogen (electronegativity 2.2), materials containing fluorine/chlorine functional groups exhibit a markedly enhanced electron transfer capability. PE showed the lowest electron transfer (1.2 nC), while FEP with the highest fluorine content reached the maximum value (9.3 nC). Furthermore, the carbon-to-fluorine ratio and molecular configurations also influenced the transferred charge. Between the fluorinated polymers ETFE and PTFE, the electron transfer increased with the carbon-to-fluorine ratio. Analysis of the structural formulas showed that ETFE and PTFE had carbon-to-fluorine ratios of 1:1 and 1:2, respectively, with the transferred charge of PTFE (7.5 nC) 1.4 times that of ETFE (5.4 nC). Although PTFE and FEP share a carbon-to-fluorine ratio of 1:2, the configurations of the fluorine-containing groups affected the transferred charge due to the presence of CF_3_ groups in FEP, whose transferred charge was 1.2 times that of PTFE. The transferred charge for PVA and PVC was slightly lower than that of the F-containing polymers, at 5.3 nC and 6.0 nC, respectively. PCTFE exhibited a transferred charge of 8.4 nC, which was much higher than that of PVC. This is because PCTFE contains not only chlorine but also fluorine functional groups, and fluorine has a higher electronegativity than chlorine.

As shown in Figure 5b, the curved contact–separation configuration consistently yielded a 20~40% enhancement in electron transfer compared to the planar configuration across the majority of polymer materials. In the curved contact–separation configuration, the relative trend in transferred charge among the various polymers remained essentially consistent with the planar configuration, and the values were generally slightly higher for all except ETFE and PCTFE. The charge values for PE, PP, PVA, PVC, PTFE, and FEP were 1.7 nC, 1.7 nC, 5.9 nC, 7.6 nC, 9.0 nC, and 10.9 nC, respectively. These correspond to 1.4, 1.2, 1.2, 1.3, 1.2, and 1.2 times their values in the planar configuration, respectively. For ETFE and PCTFE, the charge values were 5.5 nC and 8.3 nC, respectively, and their variations in their planar configurations fell within the range of measurement uncertainty. The above results verify that in the contact–separation configuration, curved contact typically generates slightly higher transferred charge compared to planar contact due to the interface curvature increasing the energy level difference between the two materials.

As shown in Figure 5c, the sliding configuration significantly enhanced the electron transfer compared to the planar contact–separation configuration, with most materials showing increases ranging from 2.4- to 29.0-fold. In this configuration, FEP had the highest transferred charge at 68.8 nC, a 7.4-fold increase over its planar contact–separation value. PCTFE increased to 67.3 nC, and ETFE increased to 61.5 nC. Notably, the charge amount for non-fluorinated materials like PVC and PP increased substantially to 52.9 nC and 40.6 nC, respectively. PP showed the most significant increase, from 1.4 nC to 40.6 nC, an approximately 29.0-fold enhancement. PE also showed a notable increase from an extremely low 1.2 nC to 30.8 nC, an approximately 26.0-fold enhancement. The enhancement for PVA was relatively limited, increasing from 5.1 nC to 12.1 nC—only 2.4 times the original value—influenced by the stickiness of this film. Overall, the sliding configuration greatly improved the electron transfer efficiency of the materials. These results show that the *Q*_sc_ of non-fluorinated polymers (PP, PE) was several times lower than that of fluorinated polymers in the contact–separation configuration. However, switching to the sliding configuration dramatically boosted the *Q*_sc_ of PP and PE, enabling them to reach a level comparable to the fluorinated polymers. Therefore, the proper selection of configurations would benefit single-electrode TENG sensing performance when the dielectric material chosen is limited.

The electron transfer properties of the eight polymers under planar, curved, and sliding configurations, as discussed above, are comprehensively summarized in Table 3. This table provides a clear comparison of the key properties and triboelectric performance of the eight polymers under different configurations.

### 3.6. Electron Transfer Characteristics of Corona-Polarized Eight Polymer Films in Three Configurations

**Figure 6:**
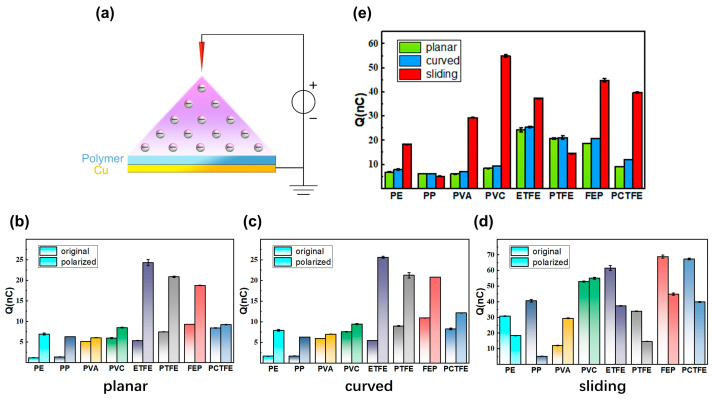
(**a**) Schematic diagram of the corona polarization setup. The white circle symbolizes the high-voltage power supply, with “+” and “–“ denoting the anode and cathode, respectively, and the red needles representing the needle electrode. (**b**–**d**) Transferred charge comparisons of polymer films before and after corona polarization under (**b**) planar contact–separation, (**c**) curved contact–separation, and (**d**) sliding configurations, respectively. Notably, under both contact–separation configurations, all polymer films exhibited increased transferred charge after corona polarization, whereas in sliding configuration, all except PVA and PVC showed decreased transferred charge post-corona polarization. (**e**) Comparison of transferred charges under three configurations after corona polarization. Post-corona polarization, six polymer films (excluding PP and PTFE) maintained higher transferred charge in sliding configuration compared to contact–separation configuration.

Corona polarization has been established as an effective method for enhancing the output performance of triboelectric nanogenerators (TENGs). To investigate whether this method exerts a universal influence on electron transfer patterns across three different configurations, the polymer films were subjected to corona polarization. A comparative analysis was then conducted on the transferred charge quantities of the three configurations after corona polarization, as well as the output characteristics of the same triboelectric systems before and after corona polarization. The experimental setup for corona polarization is illustrated in Figure 6a: the prepared sample was placed at the center of a plate electrode, above which a 7 (vertical) × 9 (horizontal) array of needle electrodes was fixed. The distance between adjacent needles was 1.2 cm vertically and 1.0 cm horizontally, with the tip of each needle positioned 15 mm above the polymer film. A high voltage of 5 kV (needles as anode, plate as cathode) was then applied for 20 min to complete the corona polarization process. This procedure ionizes the air, injects charge into the polymer film, and induces reorientation of molecular functional groups within the film, thereby modulating its triboelectric properties [38].

As shown in Figure 6b, in the planar contact–separation configuration, corona polarization significantly enhanced the transferred charge quantity (*Q*_sc_) of all polymer films, and the ranking of *Q*_sc_ among the materials also changed. After corona polarization, the three pure fluoropolymers exhibited the highest *Q*_sc_ values, with ETFE reaching the maximum of 24.3 nC, followed by PTFE and FEP at 20.8 nC and 18.8 nC, respectively. Among the chlorinated polymers, PCTFE showed a *Q*_sc_ of 9.2 nC, while PVC reached 8.5 nC. Non-halogenated polymers exhibited relatively lower *Q*_sc_ values, with PE at 6.9 nC, PP at 6.3 nC, and PVA at 6.1 nC. Compared to their original states, all materials demonstrated an increase in *Q*_sc_, with enhancement factors as follows: PE (5.9-fold), PP (4.5-fold), PVA (1.2-fold), PVC (1.4-fold), ETFE (4.5-fold), PTFE (2.8-fold), FEP (2.0-fold), and PCTFE (1.1-fold). Notably, before corona polarization in the contact–separation configuration, the *Q*_sc_ of non-fluorinated polymers (PP, PE) was several times lower than that of fluorinated polymers. However, after corona polarization, the *Q*_sc_ of PP and PE increased significantly, reaching levels comparable to those of fluorinated polymers. Meanwhile, the *Q*_sc_ ranking within the pure fluoropolymer group reversed from the pre-corona polarization order of FEP > PTFE > ETFE to ETFE > PTFE > FEP. Overall, the material performance exhibited a clear gradient: pure fluoropolymers > chlorinated polymers > non-halogenated polymers. This indicates that corona polarization is another key factor affecting the amount of electron transfer besides elemental electronegativity. As illustrated in Figure 6c, in the curved contact–separation configuration, the comparative trends of transferred charge quantity for polymer films before and after corona polarization closely aligned with those observed in the planar contact–separation configuration.

In the sliding configuration (Figure 6d), corona polarization generally reduced the transferred charge, making it different from the contact–separation configuration. Compared to the original sliding configuration, six materials showed decreases to different extents, with reduction factors ranging from 0.1 times (PP) to 0.7 times (FEP). In contrast, PVA increased significantly by 2.4 times, and PVC remained essentially unchanged (1.0 times). These differences indicate that corona polarization significantly affected electron transfer behavior under different configurations.

As illustrated in Figure 6e, the comparison of transferred charge (*Q*_sc_) under the three configurations after corona polarization reveals that the sliding configuration maintained a superiorly higher *Q*_sc_ than the contact–separation configuration for six of the eight polymer films, excluding only PP and PTFE. Furthermore, between the two contact–separation configurations, the curved configuration yielded a slightly higher *Q*_sc_ than the planar one for the majority of materials. When comparing the sliding configuration with the contact–separation configuration after corona polarization, for PVA, PVC, PCTFE, PE, FEP, and ETFE, the *Q*_sc_ in sliding configuration increased to 4.9, 6.5, 4.3, 2.7, 2.4, and 1.5 times that of the planar contact–separation configuration, respectively. This represents a notable enhancement reduction compared to the 2.4–29.0-fold enhancement observed in the original state. In contrast, for PP and PTFE, the *Q*_sc_ in sliding configuration decreased to 0.8 and 0.7 times that of the planar contact–separation configuration, respectively. Compared with the planar contact–separation configuration, the curved contact–separation configuration maintained a consistent enhancement effect across most materials after corona polarization. Specifically, the *Q*_sc_ values for PE, PVA, PVC, ETFE, FEP, and PCTFE in the curved contact–separation configuration increased to 1.1, 1.2, 1.1, 1.1, 1.1, and 1.3 times those in the planar configuration, respectively. Meanwhile, the *Q*_sc_ of PP and PTFE remained stable. These results confirm that the enhancement effect of interface curvature on electron transfer remains effective after corona polarization. 

## 4. Discussion

Our experimental results provide a comprehensive picture of electron transfer behavior in single-electrode TENGs. The discussion below delves into the implications of our key findings, which are centered on the three critical aspects of polymer functional groups, configurations, and corona polarization.

Beginning with the polymer material selection, our study clarifies the effects of functional group electronegativity, carbon-to-fluorine ratio, and molecular configuration on electron transfer. The electronegativity of polymer functional groups is a key factor influencing electron transfer. Due to the high electronegativity of fluorine, fluorinated polymers exhibit higher transferred charge in all three configurations, as clearly evidenced by the data presented in Figure 5. Furthermore, the results reveal that the carbon-to-fluorine ratio and molecular spatial configuration also significantly impact electron transfer. For instance, PTFE has a higher carbon-to-fluorine ratio than ETFE, resulting in the *Q*_sc_ of PTFE being 1.4 times greater than that of ETFE. Under the same carbon-to-fluorine ratio, FEP demonstrates higher electron transfer than PTFE due to the presence of -CF_3_ groups. Notably, for non-fluorinated polymers (PP, PE), their electron transfer in contact–separation configuration are orders of magnitude lower than in fluorinated polymers. However, a crucial finding from Figure 5b is that by switching to the sliding configuration, their performance was substantially enhanced, achieving *Q*_sc_ levels comparable to those of fluorinated polymers.

Beyond material chemistry, the choice of operational mechanics plays a decisive role. This study reveals the critical role of configuration selection on the amount of transferred charge for the same polymer–acrylic contact electrification layer pair. The sliding configuration far exceeded the contact–separation configuration in electron transfer, achieving an enhancement factor ranging from 2.4 to 29.0 times compared to the planar contact–separation configuration for eight polymer–acrylic contact electrification layer pairs. The underlying mechanism for this dramatic difference, supported by our proposed model in Figure 4c,d, lies in the transient versus continuous electron cloud overlap. Limited electron cloud overlap time at the contact interface in contact–separation configuration limits further electron transfer while continuous electron cloud overlap in sliding configuration provides more opportunities for electron transfer. Compared to the planar contact–separation configuration, the curved contact–separation configuration increased electron transfer by 20% to 40%, as shown in Figure 5a. This consistent enhancement across materials can be primarily attributed to energy level shifts in material surface states induced by interface curvature: the neutral level of convex polymers shifts downward, while that of concave acrylic shifts upward, thereby enlarging the energy level difference between the two materials and promoting more efficient electron transfer, a concept illustrated in Figure 4b.

Perhaps the most nuanced insight from this work concerns the application of corona polarization, whose effect is strongly dependent on the configuration. This study discovered significant differences in the effects of corona polarization across different configurations. The data in Figure 6c,d, demonstrate that in contact–separation configuration, corona polarization significantly enhanced the transferred charge of all polymer films. However, in the sliding configuration, the effect of corona polarization was markedly different, with Figure 6e showing that it typically reduced the transferred charge. These divergent effects indicate that applying corona polarization in contact–separation configuration is an effective strategy for optimizing device performance, but its use in sliding configuration requires careful consideration. It is noteworthy that for non-fluorinated polymers (PP, PE), whose electron transfer in contact–separation configuration is orders of magnitude lower than fluorinated polymers, corona polarization substantially enhances their performance, enabling them to achieve *Q*_sc_ levels comparable to fluorinated polymers. This interplay between material limitations and post-processing techniques opens up versatile design pathways for practical applications.

To translate these fundamental insights into practical strategies for optimizing the design of robust, real-world self-powered sensing systems, a hierarchical design framework is proposed. First, in applications where material selection is flexible, fluorinated polymers should be prioritized for their consistently high performance. When material choice is constrained—such as by cost, biocompatibility, or transparency requirements—adopting a sliding configuration can dramatically elevate the output of common polymers like PE or PP. For systems constrained to a contact-separation mode, employing a curved interface or applying targeted corona polarization offers a straightforward path to enhanced sensitivity. However, to ensure these optimized designs translate reliably from the laboratory to diverse application environments, several critical avenues for future investigation must be pursued. The performance stability under varying humidity and temperature, the systematic optimization of corona polarization parameters (voltage, duration, atmosphere), and a thorough analysis of mechanical wear and long-term durability for sliding-mode TENGs are essential next steps. Addressing these factors will bridge the gap between the fundamental electron transfer principles established here and the development of durable, high-performance, and practical TENG-based sensors for the Internet of Things, wearable electronics, and intelligent monitoring systems.

## 5. Conclusions

This study investigated three key aspects of electron transfer in eight polymer–acrylic contact electrification layer pairs to provide theoretical guidance and technical pathways for enhancing the performance of TENG-based self-powered sensors. Through a comprehensive analysis combining experiments and theoretical investigations, the impact of the polymer functional groups, the configurations of single-electrode TENGs, and corona polarization on electron transfer and the underlying mechanisms are revealed.

In summary, the main findings of this work are threefold. First, the electronegativity of polymer functional groups is a paramount factor, with fluorinated polymers exhibiting superior electron transfer ability across all configurations. Second, the selection of the configuration is critical, where the sliding configuration vastly outperforms the contact–separation configurations due to continuous electron cloud overlap, and the curved contact–separation configuration offers a slight advantage over the planar one. Third, the effect of corona polarization is highly configuration-dependent, generally enhancing performance in contact–separation configurations while reducing it in the sliding configuration.

Above all, there are some practical suggestions for the enhancement of TENG-based self-powered sensors to increase electron transfer. High-electronegativity materials, such as fluorinated polymers, are preferentially chosen for their high transferred charge in all three configurations. When the choice of triboelectric materials is limited to non-fluorinated polymers, such as polypropylene or polyethylene, employing the sliding configuration or applying corona polarization can enhance their performance to levels comparable to fluorinated materials. In scenarios where the contact–separation configuration is more applicable, curved contact interfaces should be preferentially adopted for higher electron transfer. In contact–separation configuration, corona polarization can consistently enhance device performance, whereas this technique should be applied judiciously in sliding configuration to avoid its inhibitory effects on performance.

In summary, this work provides new insights for the fabrication of high-performance self-powered sensors based on the TENG with respect to electron transfer. It establishes a comprehensive framework for optimizing device performance through the strategic selection of polymers, configurations, and corona polarization, thereby accelerating the development of self-powered systems for the Internet of Things. It also holds broad application potential in fields such as wearable electronics, environmental monitoring, and intelligent sensing, supporting the development of a new generation of highly efficient, miniaturized, and adaptive self-powered systems.

## Figures and Tables

**Table 2:** Specifications of other materials used in this study.

Material	Specification	Supplier
Copper foil	Double-sided conductive, 100 µm thick	linxin Adhesive Products Co., Ltd. (Shenzhen, Guangdong, China)
Planar acrylic plate	200 µm thick	Yongsheng Acrylic Wholesale Co., Ltd. (Suzhou, Jiangsu, China)
Curved acrylic plate	Height: 8 cm; inner radius: 9 cm; outer radius: 9.2 cm; arc angle: π/3 rad	Yongsheng Acrylic Wholesale Co., Ltd. (Suzhou, Jiangsu, China)

**Table 3:** Summary of polymer properties and transferred charge under different configurations.

Polymer	Repeat Unit Structure	C:F	Planar *Q*_sc_ (nC)	Curved *Q*_sc_ (nC)	Sliding *Q*_sc_ (nC)
PE	${-\left[CH_{2}-CH_{2}\right]}_{n^{-}}$	N/A	1.2	1.7	30.8
PP	${-[CH_{2}-CH(CH_{3})]}_{n^{-}}$	N/A	1.4	1.7	40.6
PVA	${-[CH_{2}-CH(OH)]}_{n^{-}}$	N/A	5.3	5.9	12.1
PVC	${-[CH_{2}-CHCl]}_{n^{-}}$	N/A	6.0	7.6	52.9
PCTFE	${-[CFCl-CF_{2}]}_{n^{-}}$	1:1	8.4	8.3	67.3
ETFE	${-\left[CH_{2}-CH_{2}\right]}_{m}{-[CF_{2}-CF_{2}]}_{n^{-}}$	1:1	5.4	5.5	61.5
PTFE	${-[CF_{2}-CF_{2}]}_{n^{-}}{-\left[CF_{2}-CF_{2}\right]}_{m}-$	1:2	7.5	9.0	34.0
FEP	${-\left[CF_{2}-CF\left(CF_{3}\right)\right]}_{n^{-}}$	1:2	9.3	11.0	68.8

N/A indicates not applicable for materials containing no fluorine atoms

## Data Availability

The data presented in this study can be obtained from the corresponding author upon reasonable request.
